# *WDR5* Expression Is Prognostic of Breast Cancer Outcome

**DOI:** 10.1371/journal.pone.0124964

**Published:** 2015-09-10

**Authors:** Xiaofeng Dai, Wenwen Guo, Chunjun Zhan, Xiuxia Liu, Zhonghu Bai, Yankun Yang

**Affiliations:** 1 School of Biotechnology, Jiangnan University, Wuxi, China; 2 National Engineering Laboratory for Cereal Fermentation Technology, Jiangnan University, Wuxi, China; The University of Hong Kong, CHINA

## Abstract

WDR5 is a core component of the human mixed lineage leukemia-2 complex, which plays central roles in ER positive tumour cells and is a major driver of androgen-dependent prostate cancer cell proliferation. Given the similarities between breast and prostate cancers, we explore the potential prognostic value of *WDR5* gene expression on breast cancer survival. Our findings reveal that *WDR5* over-expression is associated with poor breast cancer clinical outcome in three gene expression data sets and BreastMark. The eQTL analysis reveals 130 trans-eQTL SNPs whose genes mapped with statistical significance are significantly associated with patient survival. These genes together with *WDR5* are enriched with “cellular development, gene expression, cell cycle” signallings. Knocking down *WDR5* in MCF7 dramatically decreases cell viability, but does not alter tumour cell response to doxorubicin. Our study reveals the prognostic value of *WDR5* expression in breast cancer which is under long-range regulation of genes involved in cell cycle, and anthracycline could be coupled with treatments targeting *WDR5* once such a regimen is available.

## Introduction

The human mixed lineage leukemia-2 (MLL2) complex has been identified as a coactivator of the estrogen receptor (ER). This complex binds directly to ERα in a ligand-dependent manner through two L*XX*LL motifs in a region of MLL2 close to the C terminus [1]. Such physical interactions critically affect ER signalling, determining the central role of the MLL2 complex played in the growth of ER positive cancer cells [1]. WDR5, a member of the WD40-repeat protein family, is a core subunit of the MLL2 complex and is required for complex assembly and methyltransferase activity [2]. *WDR5* has been reported to be over-expressed in prostate cancer and is identified as a critical epigenetic integrator of histone phosphorylation and methylation, as well as a major driver of androgen-dependent prostate cancer cell proliferation [3]. It is well established that prostate and breast cancers share many similarities. Both cancers are controlled by sex hormones, which are related to hormonal carcinogenesis and oxidative DNA damage [4]. Clinically, there is a significant overlap in the age and stage at diagnosis for these two diseases. Finally, it has been reported that many of the gene pathways targeted by aberrant methylation are shared between breast and prostate cancers [5]. Given these similarities and the importance of *WDR5* in prostate cancer, we are interested in understanding the association between *WDR5* expression and some phenotypic parameters in breast cancer.

The importance of *WDR5* in breast carcinomas has recently attracted increasing attention, mostly focusing on its cooperations with immunohistochemical markers such as ER and human epidermal growth factor receptor 2 (HER2). For example, Kim et al. reported the co-activation of *WDR5* and ER signalling [6]; Yoshimaru et al. demonstrated the crucial role of the WDR5-PHB2 complex in the modulation of ER signalling [7]; and Mungamuri et al. showed the joint effort of *WDR5* silencing and chemotherapy in inhibiting the growth of HER2 positive breast tumour cells [8].

In this study, we are interested in understanding the potential prognostic value and the regulatory mechanism of *WDR5* expression for breast cancer survival, particularly for patients receiving the anthracycline regimen (a chemotherapy commonly used in breast cancer). Furthermore, gene silencing and drug treatment experiments were conducted using 4 breast tumour cell lines to validate the findings.

## Data and Materials

### Data

#### Gene expression data

The gene expression data used in this study are summarized in Table 1.

**Table 1:** Description of the data sets used for gene expression survival analysis.

Data source	GSE1456	GSE4922	GSE24450
**Sample size**	159	249	183
**Event**	40	89	39

**The GSE24450 data set** consists of 183 primary breast tumour samples (including 39 cases of breast cancer specific death or distant metastasis), among which 151 were collected as a part of the unselected series at the department of Oncology of the Helsinki University Central Hospital (HUCH) in 1997, 1998 and 2000 [9,10] and at the department of Surgery from 2001 to 2004 [11]. The remaining 32 patients belong to an ongoing collection of the additional familial breast cancer series from the department of Clinical Genetics at HUCH. Among these patients, 68 are known to have received anthracycline regimen (including 18 events), and 23 are not (including 9 events). These samples comprise 80 luminal A, 12 luminal B, 8 HER2 positive, 11 basal, 7 triple negative tumours, as well as 65 samples without subtype classification.

Total RNA was extracted from the 183 primary breast tumours, and the samples were processed and hybridized to Illumina HumanHT-12_V3 Expression BeadChips, containing 24660 Entrez Gene entities according to the manufacturer recommendations (http://www.illumina.com). Gene expression profiling was carried out at SCIBLU Genomics Centre, Lund University, Sweden. Raw microarray data was imported into R [12] and processed by the methods included in the BioConductor facilities [13,14]. Briefly, after quality control [15], the data was normalized using the quantile method [16] and the gene expression matrix was obtained by averaging the probes mapped to the same Entrez Gene IDs [17].

**The GSE1456 data set** (GPL97) was retrieved from Gene Expression Omnibus (GEO) [18], which comprises 159 samples (including 40 relapse or breast cancer specific death) [19]. Tissue material was collected from all breast cancer patients that received surgery at Karolinska Hospital (Stockholm, Sweden) from 1^st^ January 1994, to 31^st^ December 1996 and were identified in the Stockholm-Gotland breast cancer registry [19]. RNA was extracted according to the RNeasy mini protocol (Qiagen, Hilden, Germany) [19]. All tumour specimens were profiled on Affymetrix Human Genome U133A arrays at Bristol-Myers Squibb (Princeton, New Jersey) [19]. Data pre-processing includes normalization (using the global mean method), natural-log-transformation and scaling (i.e., adjusting the mean signal to a target value of log 500) [19]. The maximum follow-up time is 102 months. The relapse free survival (RFS) and overall survival (OS) were studied, depending on their availability. These samples include 39 luminal A, 23 luminal B, 15 HER2 positive, 25 basal, 37 normal-like tumours and 20 with unknown subtype classification.

**The GSE4922 data set** (GPL97) was retrieved from GEO [18], which is comprised of 249 samples (including 89 events with relapse or breast cancer specific death) [20]. Tissue samples were collected in Uppsala County, Sweden, from 1^st^ January 1987, to 31^st^ December 1989 [20]. RNA was extracted using the RNeasy mini protocol (Qiagen, Hilden, Germany), and the tumour samples were profiled on the Affymetrix U133A genechips at the Genome Institute of Singapore [20]. The data were normalized using the global mean method, natural-log-transformed and scaled by adjusting the mean signal to a target value of log 500 [20]. The maximum follow-up time is 153 months. DFS was analysed here, depending on its availability. These samples comprise 211 ER positive, 34 ER negative tumours and 4 samples with ER positivity unspecified.

**BreastMark** is an online tool for examining the prognostic value of putative genes in breast cancer, which integrates gene expression and survival data from 26 datasets on 12 different microarray platforms. These correspond to approximately 17000 genes in up to 4738 samples. DFS is analysed and the median is used to dichotomize the data. There are 1378 samples with information available for the gene *WDR5*, including 402 luminal A, 497 luminal B, 175 HER2 positive, 254 basal tumours and 50 samples without subtype specification. 11 datasets were included in this analysis given their information available on *WDR5*. These datasets are Desmedt et al., 2009.; Bos et al., 2009.; Buffa et al., 2011.; Calabro et al., 2009.; Loi et al., 2008.; Heikkinen et al., 2011.; Hu et al., 2006.; Kok et al., 2009.; Chang et al., 2005.; Sabatier et al., 2010.; and Sircoulomb et al., 2010 in [21].

#### Genotype data

**The TCGA data set**, comprised of the primary solid tumour genotype data retrieved from the TCGA portal at http://tcga.cancer.gov/dataportal, was used together with the gene and protein expression data of the same set of samples for the eQTLs analysis. There were 502 and 385 samples, shared between the genotype and the gene and protein expression data, respectively. The TCGA genotype data was produced using the Affymetrix GenomeWide Human SNP array 6.0, which includes 906600 SNPs. The raw data was processed using the birdseed algorithm, which uses a customized expectation-maximization (EM) method to fit two-dimensional Gaussians to SNP data and generates the genotypes and confidence scores for each sample and each SNP. Genotypes with confidence score above 0.1 were coded as missing data [22,23] in the analysis. The data was retrieved on 15th January, 2013.

### Materials

#### Cell culture

Four breast cancer cell lines MCF7 (ATCC No. HTB-22), MDAMB361 (ATCC No. HTB-27), MDAMB231 (ATCC No. HTB-26) and HCC1937 (ATCC No. CRL-2336) were used in the experiments (S1 Table). Cells were mycoplasma tested and verified by sequencing.

MCF7 cells were cultured in DMEM medium (Gibco) supplemented with 10% fetal bovine serum (Gibco), 1% glutamine (Thermo Scientific), 1% penicillin-streptomycin (Gibco), and 10 μg/ml insulin (Sigma). MDA-MB-361 and MDA-MB-231 cells were cultured in DMEM medium (Gibco) supplemented with 10% fetal bovine serum (Gibco), 1% glutamine (Thermo Scientific), and 1% penicillin-streptomycin (Gibco). HCC1937 cells were cultured in RPMI1640 medium (Gibco) supplemented with 10% fetal bovine serum, 1% glutamine, and 1% penicillin-streptomycin. Assay ready cells were prepared by culturing cells in a large batch and aliquoting them into ampules that were kept in liquid nitrogen in solution containing 90% FBS and 10% DMSO. Immediately prior to transfection, cells were thawed and washed with culture medium and cell number was counted using a hemocytometer.

#### Drugs

Doxorubicin, an anthracycline antibiotic, was ordered from Sigma-Aldrich (catalogue no. D1515) and used in drug screen.

#### Controls

Qiagen AllStars Hs Cell Death Control (catalogue number: SI04381048) and Ambion Silencer Select Negative Control (catalogue number: 4390844) were used as the positive and negative controls, respectively.

#### siRNAs

Eight small interfering RNAs (siRNAs) targeting WDR5 were used, including 4 ordered from Ambion (s21862, s21863, s21864, s225470) and 4 from Qiagen (SI5128767, SI00118916, SI00118923, SI00118909).

## Method

### Gene expression survival analysis

The survival analysis on expression of the gene *WDR5* was carried out using the GSE24450, GSE1456 and GSE4922 data sets. The median was used to split gene expression data into high and low expression. The gene expression survival analysis was conducted using the log-rank test, and the p values from the chi-square test were used to assess the statistical significance. The DFS was analysed for all datasets, where the maximum follow-up times are 5 years, 8.5 years and 12.75 years in GSE24450, GSE1456 and GSE4922 data, respectively. We also examined the 10-year breast cancer specific death using GSE24450. Subgroup analyses using anthracycline treated and non-treated samples were conducted using GSE24450 data, given its chemotherapy treatment information.

Additionally, BreastMark was employed to examine the association between *WDR5* gene expression and breast tumour clinical outcome. In addition, such analysis was conducted in luminal A and luminal B tumours. It treats each of the 26 datasets separately when determining which group a sample belongs to in order to negate study-specific effects. The datasets are combined and a global pooled survival analysis is performed. Survival curves are provided based on Kaplan-Meier estimates, the survival difference is shown by the log-rank p value, and the hazard ratio is computed using Cox regression analysis [21].

### eQTL SNP survival analysis

The primary solid tumour genotype and level 3 gene expression data were used for the eQTL analysis. TCGA copy number variation (CNV) data, retrieved using cBio cancer genomics portal (http://www.cbioportal.org/public-portal/) [24], was used as the covariate. In total, 502 samples which have genotype, gene expression and CNV data available, were used in the analysis. The eQTL analysis (linear model was applied) was carried out with and without CNV as the covariate, with SNPs having p-values no greater than 0.01 being selected.

The tagging SNPs were retrieved using SNAP (Proxy Search) [25], where Caucasion samples (CEU) from 1000 Genomes Pilot 1 were used as the data set with the distance limit and *r* ^2^ set to 500 and above 0.8, respectively. The retrieved SNPs as well as their tagging SNPs were mapped to genes using GRAIL (beta) [26]. In GRAIL, the CEU samples from HapMap release 21 or Human Genome Assembly 17 was used, and PubMed December 2006 was used as the ‘Functional Datasource’., and ‘Gene Size Correction’ was set as ‘on’. The genes significantly associated with the SNPs (GRAIL p value < 0.05) were firstly selected, and those whose expression significantly (p value < 0.05) associated with breast tumour survival according to BreastMark were finally selected.

Consequently, *WDR5* eQTL SNPs having significant association with these genes were selected. The tagging SNPs were checked among these SNPs using SNAP (Pairwise LD) [25] with the same parameter setting as when retrieving the tagging SNPs.

### Gene network analysis

The genes significantly associated with these *WDR5* eQTL SNPs also significantly affect breast tumour survival, indicating the networking of these genes with *WDR5* as well as the consensus pathways they involve.

The network analysis was conducted among these eQTL SNP associated genes and *WDR5* using the Ingenuity Pathway Analysis (IPA) tool (Ingenuity Systems, www.ingenuity.com). The number of molecules shown in the network was set to a default limit of 35, i.e., only the most important genes with the maximum connectivity were included. The resulting networks were scored by Fisher’s exact test and the most significant (having the highest IPA score) network was selected.

### Experimental validation

#### Experimental design

The drug response of two luminal breast cancer cell lines, i.e., MCF7, MDAMB361, and two non-luminal cell lines, i.e., MDAMB231 and HCC1937, to the treatment of doxorubicin were conducted. Eight concentrations, i.e., 1 nM, 10 nM, 25 nM, 100 nM, 250 nM, 1000 nM, 2500 nM, 10000 nM were used. Eight siRNAs, 4 from Ambion and 4 from Qiagen, were designed with 5 replicates. The positive control was Qiagen AllStars Mm/Rn Cell Death Control (catalog number: SI04939025) with 12 replicates, and the negative controls were Ambion Silencer Select Negative Control (catalog number: 4390843) and Qiagen AllStars Negative Control (catalog number: 1027281) for siRNAs ordered from each company with 32 replicates, respectively. The Ambion positive control was not used given its unstable performance tested during optimization. Negative-plus-drug controls (i.e., negative controls treated with drug at each concentration) were included as well, each having 12 replicates. Also included in each plate were 76 wells of empty cells.

#### Experimental procedure

A custom human siRNA library was acquired from Qiagen and Ambion (Silencer Select) on 384-well plates. Library and control siRNAs were transferred to black clear bottom tissue-culture treated 384-well plates (Corning #3712) using the acoustic droplet ejection method with the Echo 550 liquid handler (Labcyte). The assay plates were kept sealed in -20°C until used. Prior to transfection, 5 μl of Optimem medium (Gibco) containing 75 nl (MCF7) or 50 nl (MDA-MB-361, MDA-MB-361 and HCC1937) of Lipofectamine RNAiMAX (Invitrogen) was added per well using Multidrop Combi nL (Thermo Scientific) and plates were mixed for 15–120 min. After mixing, 500 cells in 20 μl of culture medium were added per well using Multidrop Combi (Thermo Scientific). Final concentration of siRNA in assay plates was 10 nM. After transfection, cells were incubated at 37°C for 4 days in the presence of 5% CO2 in a cell incubator (HERACell 240, Thermo Scientific). Doxorubicin (Sigma) was added to transfected cells 24 h after transfection. Doxorubicin was delivered with acoustic dispensing into a Matrix 384 cone bottom plate (Thermo Scientific) and dissolved into media. The dissolved drug was then pipetted onto transfected cells using Biomek FxP (Beckman Coulter). Cell proliferation was measured 96 h after transfection by adding 25 μl per well of CellTiter-Glo (Promega), followed by shaking for 5 min at 600 rpm (Titramax 1000, Heidolph), centrifugation for 5 min at 1000 rpm (SL40R, Thermo Scientific), and luminescence was detected using Pherastar FS plate reader (BMG Labtech).

#### Data processing

To assess the quality of the cell viability assay, Z factors were calculated for each cell line to measure the effect size between negative and positive controls [27].

To evaluate the effect of each siRNA on baseline cell viability, the raw intensities of siRNA-transfected wells at the lowest drug concentration were compared with cells without transfection for each cell line. The p-values were computed as the two-tailed probability at 95% confidence from a standard normal distribution.

The dose-response curve of doxorubicin treatment was obtained for each siRNA using the ‘drc’ package [28] in R, where a four parameter log logistic model (LL.4) was used for data fitting. Corresponding half-maximum inhibitory concentration (IC50) values were produced.

## Results

### Gene expression survival analysis

The association of *WDR5* gene expression with patient survival was analysed using GSE24450, GSE1456 and GSE4922 data sets (Table 1). In addition, using the chemotherapy treatment information available in GSE24450, we conducted survival analysis in anthracycline-treated and-untreated groups.

**Fig 1:**
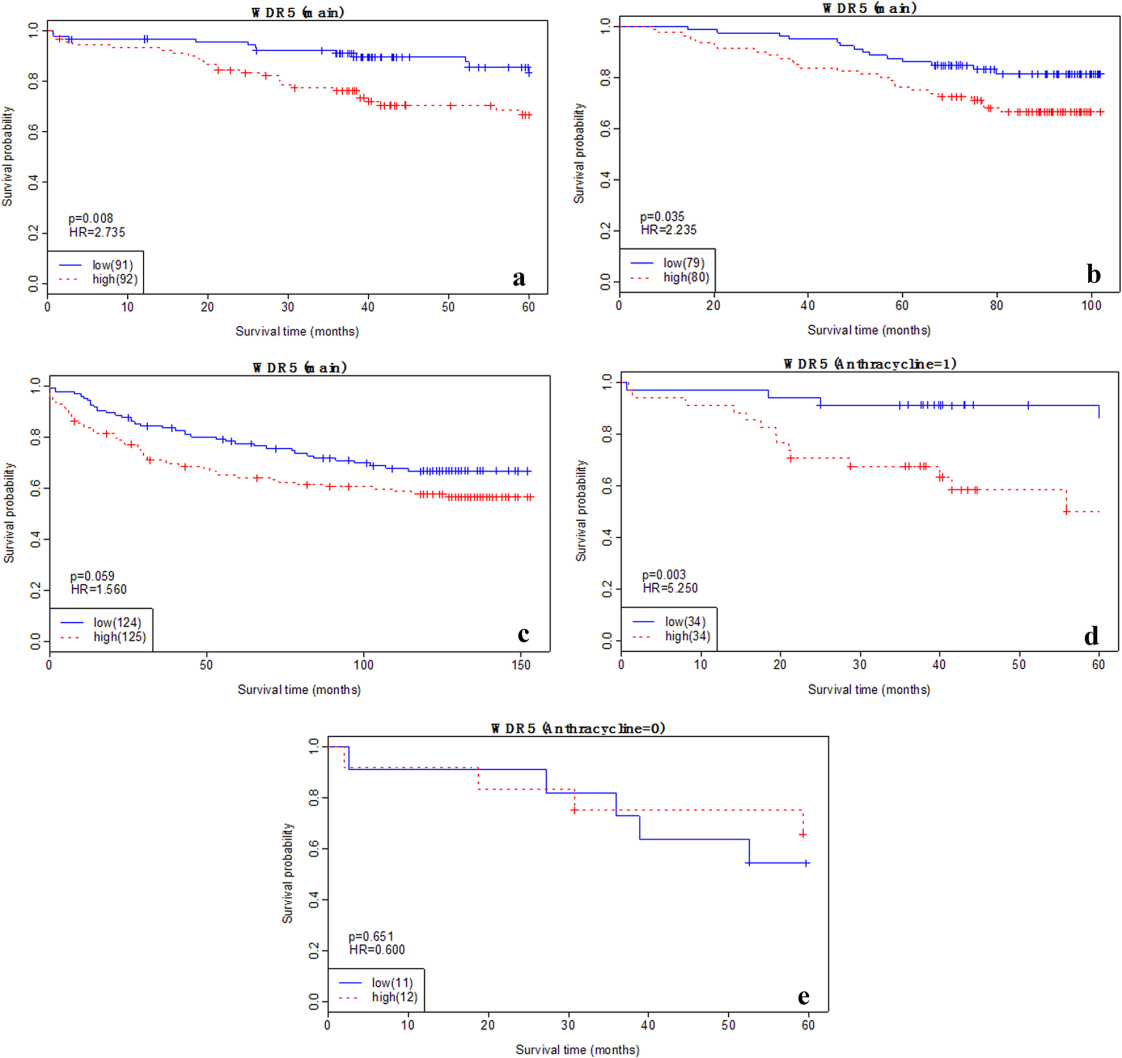
Kaplan-Meier plots of breast cancer patient survival based on WDR5 expression. Plots represent DFS from the main analysis of A) GSE24450, B) GSE1456, and C) GSE4922 data sets, and subgroup analysis of D) anthracycline treated and E) untreated tumours from GSE24450 data. The p value and hazard ratio (HR) are shown in each subplot. The number of patients is shown in the brackets in the legends. Data on the breast cancer specific death over 10 years using GSE24450 are shown in S1 Fig.

At the transcriptional level, higher *WDR5* expression shows consistent association with poorer breast cancer disease-free survival (DFS) across the three tested data sets (Table 2). In GSE24450, the hazard increase associated with *WDR5* over-expression is amplified in anthracycline-treated subgroup, where the p value decreases from 0.008 to 0.003 and the hazard ratio (HR) increases from 2.74 to 5.25 (Fig 1A and 1D); while in the untreated subgroup, no significant result is obtained (Fig 1E: p = 0.651, HR = 0.6). The association of *WDR5* over-expression with 10-year breast cancer specific death is less significant than with DFS using the GSE24450 data. As can be seen from the S1 Fig, 10-year breast cancer specific survival patterns are similar to DFS for all analyses (total or treatment-specific) (Fig 1A, 1D and 1E); however, statistical significance is achieved only in anthracycline-treated tumours (p = 0.027, HR = 4.306). The prognostic value of *WDR5* over-expression is also conferred by data sets GSE1456 and GSE4922, where significantly reduced survival (p = 0.034, HR = 1.98) is observed using GSE1456, and the marginal significance (p = 0.059, HR = 1.56) is obtained using GSE4922.

**Table 2:** Summarized statistics of the association between *WDR5* gene expression and breast cancer patient survival.

Data set	Subgroup	P	HR	Sample
**GSE24450**	Main	0.008	2.74	183 (39)
**GSE1456**	Main	0.034	1.98	159 (40)
**GSE4922**	Main	0.059	1.56	249 (89)
**GSE24450**	Anthr+	0.003	5.25	68 (18)
**GSE24450**	Anthr-	0.651	0.60	23 (9)

The p value and hazard ratio (HR) of tumours over-expressing *WDR5* are depicted below. In the ‘Subgroup’ column, ‘Anthr+’ and ‘Anthr-’ labels represent the anthracycline treated and untreated group respectively, and ‘main’ means all samples are used in the analysis. The number of patients is shown in the ‘Sample’ column, with the number of events listed in the brackets.

**Fig 2:**
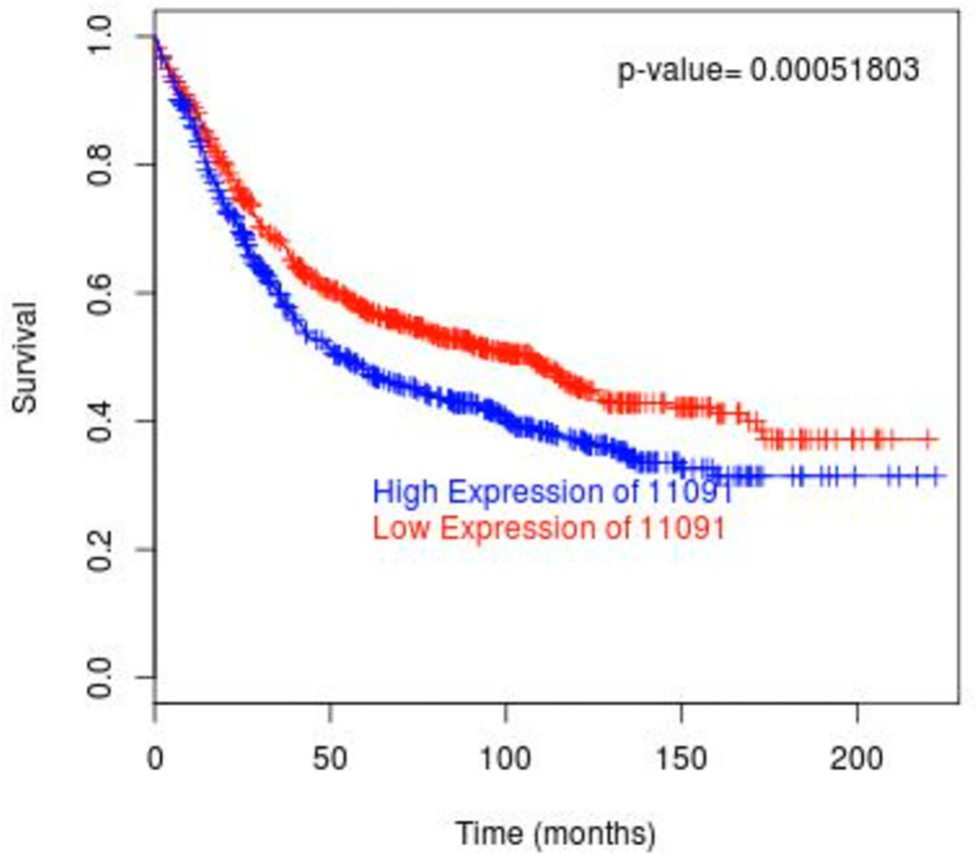
Kaplan-Meier plot of breast cancer patient survival based on *WDR5* expression using MTCI BreastMark. Plots represent the DFS. n = 1378, number of events = 715, Hazard ratio = 1.297 (1.119–1.502), score (log rank) test = 12.03 on 1 df, p = 0.0005236.

The result of the DFS analysis using BreastMark [21] is consistent with those obtained from the aforementioned individual datasets (Fig 2). Specifically, 1378 samples from 11 datasets were analysed including 715 events from BreastMark. High expression of *WDR5* (blue curve) is significantly associated with decreased survival (p = 0.00051803, HR = 1.297). Given the large sample size and comprehensive information on subtype classification in BreastMark, we also conducted the DFS analysis in luminal A and luminal B tumours (both are ER positive) as the MLL2 complex (comprising *WDR5*) is known to critically affect ER signalling [1]. However, no significant association was found between *WDR5* expression and clinical outcome in neither subtype (S2 and S3 Figs).

### eQTL SNP survival analysis

From SNPs available in TCGA, 19471 were found to be associated with *WDR5* expression, with 14493 present in GRAIL and mapped to 523 genes with statistical significance. Among these genes, 130 were significantly associated with breast tumour patient survival, which correspond to 130 eQTL SNPs that are not under linkage disequilibrium. 70 out of these SNPs were revealed using TCGA data regardless of whether CNV is adjusted, 51 were found without removing the confounding effect of CNV, and 9 were uncovered with CNV being adjusted. The statistics of these eQTL SNPs were summarized in S2 Table.

We further checked the chromosome locations of the eQTL SNPs (S4 Fig). The 130 SNPs are spread across all human autosomes, with the majority (approximately 18% SNPs) located on chromosome 1. Three SNPs are located on the same chromosome with *WDR5*, with a distance of around 38.8 Mb (rs7860361) and 108.9 Mb (rs4242698) and 130.5 Mb (rs16923216) away from the gene, respectively.

**Fig 3:**
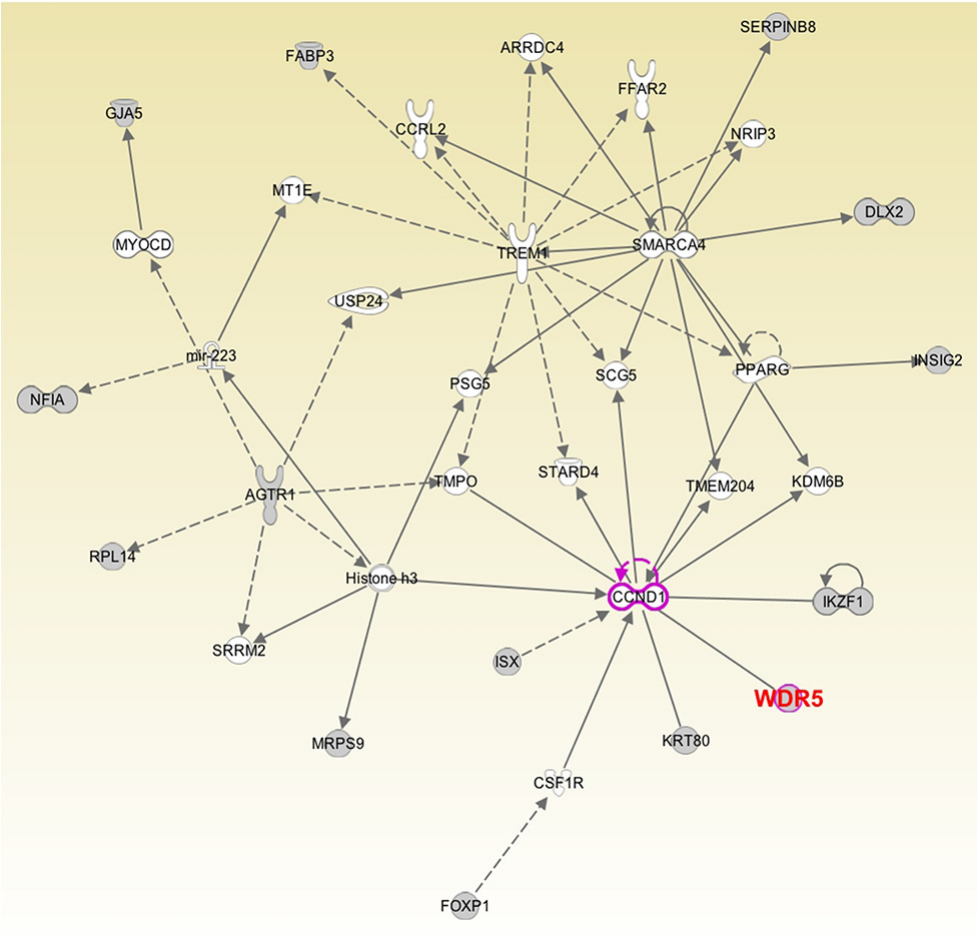
The top network of *WDR5* eQTL associated genes predicted using IPA. 35 components were chosen to be included. Genes in gray are *WDR5* and its eQTL associated genes, and the rest are genes closely related to them.

These eQTL SNPs are mapped to 130 genes (S2 Table). The top network involving *WDR5* (produced using IPA) is “Cellular Development, Gene expression, Cell Cycle” which is scored 40 (Fig 3). The “STAT3” and “HIF1α” signalings popped up in the top canonical pathways, with the p values being 9.53E-06 and 5.76E-05, respectively (S5 Fig).

### Experimental validation

The Z factor (effect size between positive and negative controls) [27] was computed to assess the screen assay quality, with Z≥0.5 indicative of an excellent assay, 0≤Z<0.5 suggestive of a marginal screen quality, and Z<0 indicative of too much overlap between positive and negative controls for the assay to be useful. The Z factor averaged across cell lines using drug-free negative controls is 0.6 (S3 Table). Most negative Z factors were shown for assays using negative-plus-drug at 1000 nM, including MCF7 Qiagen, MDA231 Ambion, MDA361 Ambion, MDA361 Qiagen, HCC1937 Ambion; or mostly occur in MDA231 cell line including Ambion 1000 nM, Ambion 2500 nM, Ambion 10000 nM, Qiagen 2500 nM, Qiagen 10000 nM (S3 Table).

**Fig 4:**
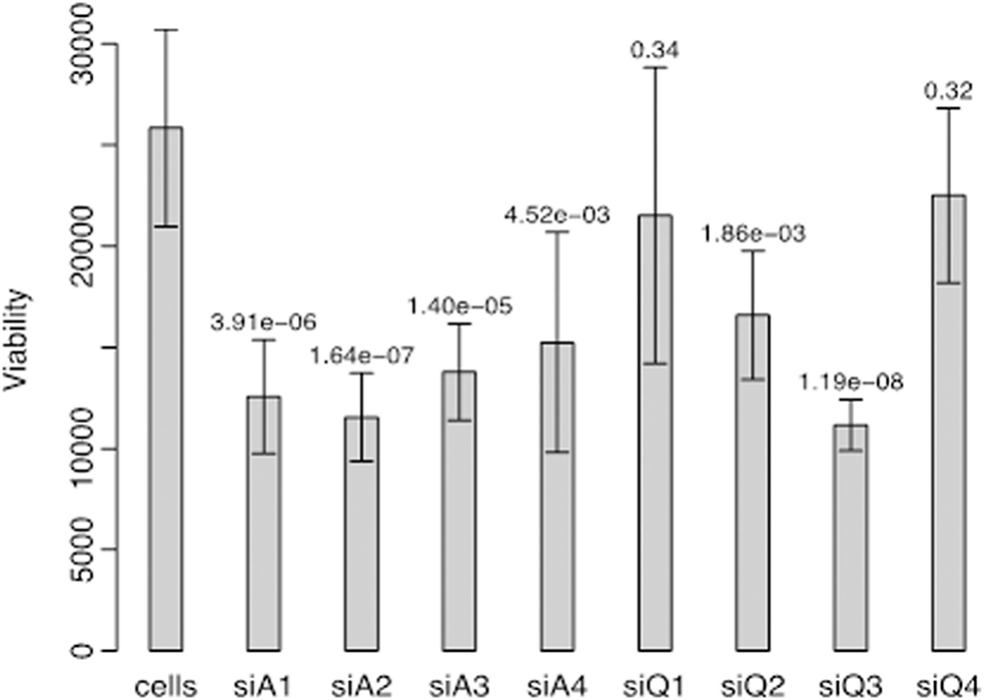
Boxplots showing the effect of *WDR5* knockdown on cell viability in the MCF7 breast cancer cell line.

The cell viability dramatically decreases when *WDR5* is knocked down in MCF7 cells, with 6 out of 8 siRNAs reaching statistical significance (Fig 4). Such an observation was not consistently significant in the other tested cell lines (S6 Fig).

Doxorubicin was applied to siRNA-transfected MCF7 cells to examine whether the association between *WDR5* over-expression and survival is related to treatment. This is important to address since anthracycline-related regimens are the most common forms of adjuvant chemotherapy in breast cancer currently. IC50 values do not significantly differ between siRNA-transfected cells and controls, suggesting that *WDR5* deficiency does not affect the drug response of breast tumour cells to doxorubicin (S7 Fig).

## Discussion

At the genetic level, we find 130 SNPs affecting *WDR5* expression, including 3 (rs7860361, rs4242698 and rs16923216) distant *cis* eQTLs and 127 *trans* eQTLs. Long-range regulatory elements are reported to constitute an important mechanism for gene regulation. Recent studies have identified several genes that under long-range regulation during breast cancer progression, including those encoding transcription factors such as ER [29,30], PR [31], AP1 [32], AP2 [33], FoxA1 [34,35], GATA3 [36,37], architectural components such as cohesion [38,39] and SATB1 [40,41], coactivators such as p300/CBP [42,43] and SRC1-3 [44]. As a core subunit of MLL and SET1 histone H3K4 methyltransferase complexes, WDR5 is required for complex assembly and methyltransferase activity [2], which may be a newly identified coactivator whose expression is under long-range regulation during breast tumour progression.

The genes significantly associated with these *WDR5* eQTL SNPs are shown to affect patient survival with statistical significance. These genes, together with *WDR5* are densely inter-connected by many genes involved in cellular development, gene expression, and cell cycle. For example, CCND1, which directly interacts with WDR5, is characterized by a dramatic periodicity in protein abundance throughout the cell cycle; and TP53 is known to regulate cell cycle and functions as a tumour suppressor. These genes imply several important cancer core signallings, such as cell cycle, PI3K, Wnt, and NFκB. Also, *WDR5* and these eQTL associated genes are enriched in STAT3 and HIF1α signalings, suggesting the potential role of *WDR5* on cell proliferation and angiogenesis. ER is also present as a core protein in the network (Fig 3), complying with the fact that MLL2 complex is a coactivator of ER [1].

In the experiments, we find that knocking down *WDR5* in MCF7 dramatically suppresses its expression. MCF7 has been previously applied with a success to explore the effect of MLL2 depletion on breast tumour cell growth [1], and the association of reduced breast tumour cell proliferation with *WDR5* deficiency has been previously demonstrated using western blot in MCF7 [6]. Our observations in MCF7 confirm previous studies at the transcriptional level and recapitulate our findings in the survival analysis, implying an association between low *WDR5* expression and good prognosis via reduced tumour cell viability. We did not observe significant cell reduction in MDAMB231, HCC1937 and MDAMB361 after transfecting cells with *WDR5* siRNAs. Unlike MCF7, these three cell lines all harbour *p53* mutations, which may explain the unreduced viability of *WDR5*-deficient tumour cells. Alternatively, these results may suggest a subtype specific association, given that MCF7 is ER+HER2-, MDAMB361 is ER+HER2+, and MDAMB231 and HCC1937 are basal cell lines (ER-HER2-).

We find from our computational analysis that low *WDR5* expression is associated with improved clinical outcome under anthracycline treatment, while the *in vitro* study reveals no improved sensitivity to this drug after knocking down *WDR5*. This seemingly inconsistency may suggest that, while low *WDR5* expression is associated with improved sensitivity to anthracycline a baseline expression of *WDR5* is needed to have such a synergistic effect with this drug. Alternatively, this may be a result of the differences between cell lines and tumour cells, where although cell lines have been widely used for studying tumour cell behaviour *in vitro*, some features could not be well captured by them, especially for the loss of certain cellular signallings due to the removal of ancillary cells such as fibroblasts from the cell culture.

We focused on the prognostic value of *WDR5* expression on breast tumour survival in this study, and particularly analyzed ER positive tumours in BreastMark. Though *WDR5* expression level was significantly associated with clinical outcome, such an association vanished when analyzing ER positive tumours alone in BreastMark. However, significant association was revealed using the GSE4922 dataset which is predominantly composed of ER positive tumours (221 out of 249 tumours). Besides, it is reported that the transactivation of *WDR5* activates ER signalling in breast cancer cells [6], and the *WDR5-PHB2* complex has a crucial role in the modulation of ER signalling in breast cancer cells [7]. Though we could not exclude the possibility that *WDR5* expression is not a good prognostic marker in ER positive breast tumours, we should keep in mind that BreastMark uses PAM50 for tumour classification which is based on gene expression profiling but not immunohistochemical markers and may fail in reflecting the involvement of ER in such an association. We did not conduct the survival analysis in ER positive tumours using GSE24450 and GSE1456, because they do not have sufficient sample size (92 samples in GSE24450, 62 samples in GSE1456) or events (17 events in GSE24450, 18 events in GSE1456) to allow statistically sound analysis after removing cases with unspecified subtypes (65 cases in GSE24450, 20 cases in GSE1456) and ER negative tumours.

In our next step, we will explore the prognostic value of *WDR5* in ER positive tumours using more datasets encompassing sufficient tumour samples. Also, we will investigate whether *WDR5* expression affects the response of cells to hormone treatment, for which experimental approaches such as studies on tamoxifen-treated ER positive breast tumour cells before and after knocking down *WDR5* would be appropriate. Alternatively, as the genes encoding the other components of the MLL2 complex (MLL2, ASH2, RBQ3) are also reported to be amplified in some cancers [45–48], we could investigate their prognostic value and potential interactions with *WDR5* during breast carcinogenesis. Further, It would be interesting to test our findings *in vivo* using, e.g., mouse xenografts.

## Conclusions

*WDR5* has been previously reported to be overexpressed in prostate cancer and *WDR5* expression is critical for proliferation of tumour cells [3]. Here, by studying the association between *WDR5* expression and breast cancer outcome using three independent data sets, we find that high levels of *WDR5* is prognostic of poor breast cancer survival. An ensemble survival analysis using BreastMark [21] confirms our results at the transcriptional level. Analysis at the genetic level reveals that *WDR5* expression is under long-range regulation of genes involved in cellular development, gene expression and cell cycle, confirming with its proliferative roles in carcinogensis observed at the gene expression level. Gene knockdown experiments show that *WDR5* is important for breast tumour cell proliferation in MCF7, providing additional support for our findings and suggesting the involvement of other factors such as p53, ER, HER2 in such an observation. In addition, lacking *WDR5* expression does not induce tumour cell resistance to doxorubicin, allowing its combined usage with traditional chemotherapy, e.g. anthracycline, once such a regimen is available.

Our study reveals the prognostic value of *WDR5* expression in breast cancer, which is a potential diagnostic marker in clinical practice. *WDR5* expression is under long-range regulation of genes involved in cellular development, gene expression and cell cycle. Also, we propose *WDR5* as a potential drug target for breast cancer treatment which is combinable with traditional regimen such as anthracycline.

## Supporting Information

S1 Fig Breast cancer-specific death over 10 years using GSE24450 data. (DOCX)

S2 Fig Kaplan-Meier plot of Luminal A breast tumour patient survival based on *WDR5* expression using MTCI BreastMark. Plots represent the DFS. n = 402, number of events = 150, Hazard ratio = 1.034 (0.7383–1.447), Score (log rank) test = 0.04 on 1df, p = 0.84585.
(DOCX)

S3 Fig Kaplan-Meier plot of Luminal B breast tumour patient survival based on *WDR5* expression using MTCI BreastMark. Plots represent the DFS. n = 497, number of events = 290, Hazard ratio = 1.167 (0.9273–1.47), Score (log rank) test = 1.74 on 1df, p = 0.1871.
(DOCX)

S4 Fig Histogram of the chromosomes locating the eQTL SNPs of *WDR5*. *WDR5* is located on chromosome 9 as shown in red.
(PDF)

S5 Fig The canonical networks of the *WDR5* eQTL associated genes predicted using IPA. (JPG)

S6 Fig Boxplots showing the effect on cell viability after knocking down *WDR5* in different breast cancer cell lines. (TIF)

S7 Fig Drug response curves (DRC) of breast tumour cells (MCF7) treated with doxorubicin after *WDR5* knockdown using different siRNAs. The siRNA codes are shown in brackets.
(DOCX)

S1 Table Cell line properties and culture medium. (XLSX)

S2 Table Detailed statistics of the uncovered *WDR5* eQTL SNPs and the associated genes. ‘eQTL SNP’ lists the SNPs whose expression significantly associated with *WDR5* expression; ‘eQTL source’ shows the data used for the eQTL analysis, with ‘TCGA’ and ‘TCGA_coCNV’ each representing the usage of TCGA data without and with CNV being adjusted, respectively; ‘eQTL p_TCGA’ and ‘eQTL p_TCGA_coCNV’ each shows the eQTL p value without and with CNV being adjusted using TCGA data, respectively. ‘Gene’ lists the genes mapped to these eQTL SNPs with statistical significance by GRAIL, with the GRAIL p value showing in ‘GRAIL p’. ‘BM p’ shows the p value from BreastMark in survival analysis of the corresponding gene; ‘BM ratio’ lists the hazard ratio in the survival analysis for each gene; ‘BM n’ and ‘BM events’ are the number of patients and events, respectively, used in the survival analysis. ‘Chromosome’ is the chromosome where the corresponding gene and SNP are located.
(XLSX)

S3 Table Z factors for each assay. 'Negative Concentration' shows the concentration at which negative Z factor is observed.
(XLSX)
