# Supplementary figures and images for: WDR5 Expression Is Prognostic of Breast Cancer Outcome
Source: PLoS One. 2015 Sep 10;10(9):e0124964. doi: 10.1371/journal.pone.0124964 (PMC4565643; doi:10.1371/journal.pone.0124964)

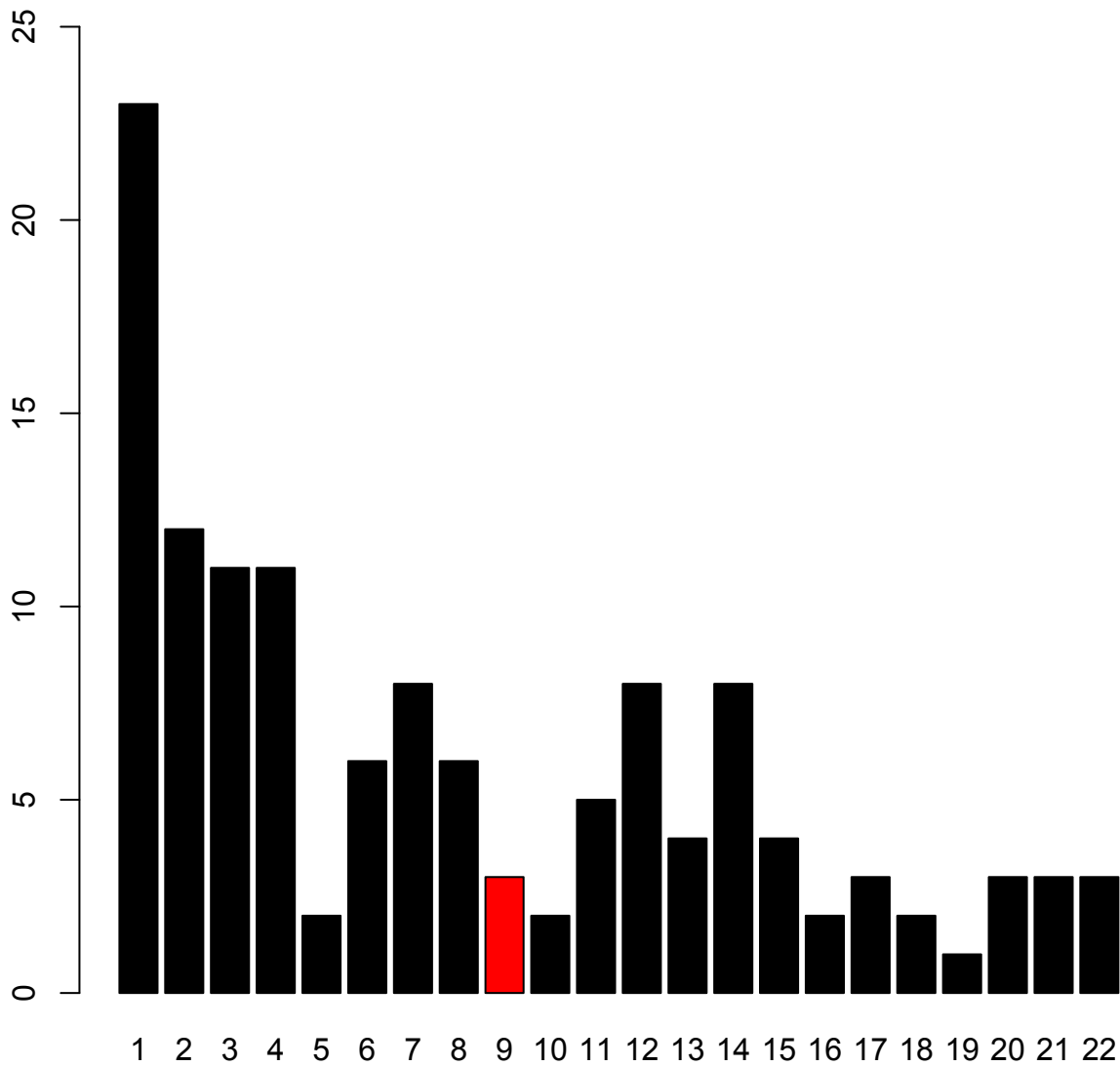

Supplement: S4 Fig — WDR5 is located on chromosome 9 as shown in red. (PDF) [file pone.0124964.s004.pdf]

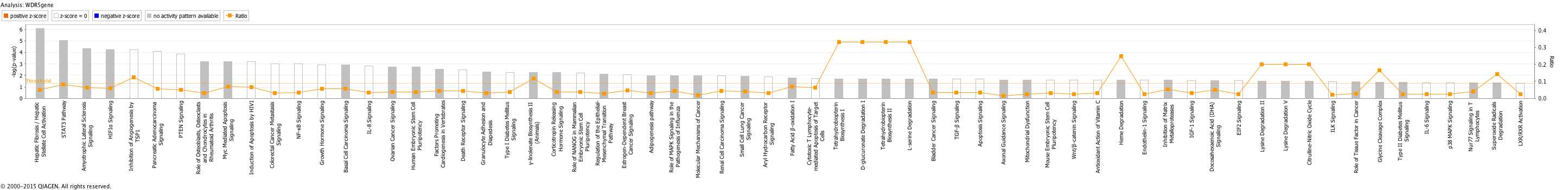

Supplement: S5 Fig — (JPG) [file pone.0124964.s005.jpg]

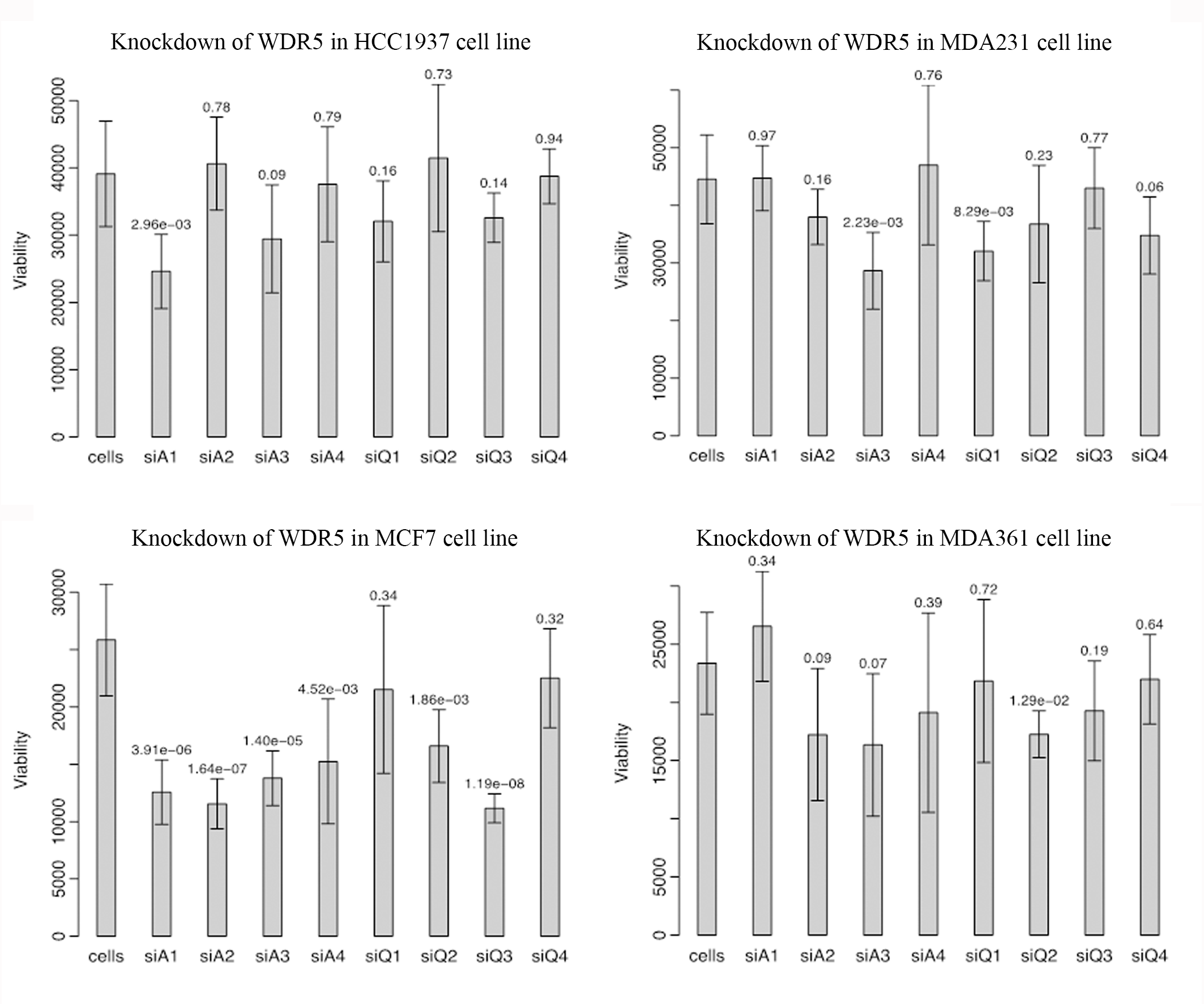

Supplement: S6 Fig — (TIF) [file pone.0124964.s006.tif]
