# Supplementary material for: WDR5 Expression Is Prognostic of Breast Cancer Outcome
Source: PLoS One. 2015 Sep 10;10(9):e0124964. doi: 10.1371/journal.pone.0124964 (PMC4565643; doi:10.1371/journal.pone.0124964)
Supplement: Supplementary file 2 [file pone.0124964.s001.docx]

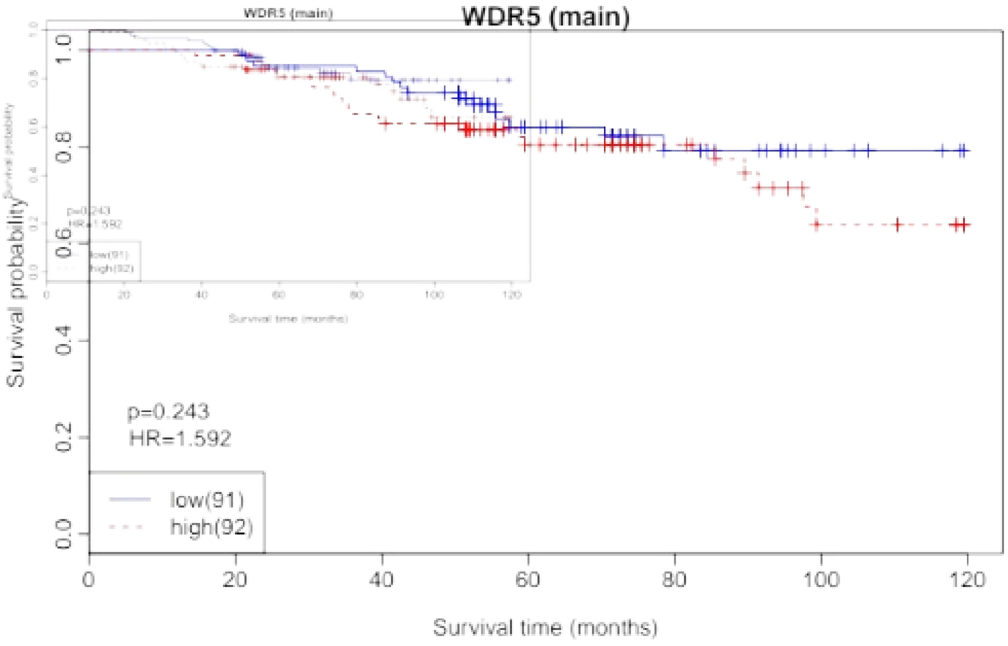
WDR5 (main)
Survival probability
1.0
0.8
0.6
0.4
0.2
0.0
p=0.243
HR=1.592
low(91)
high(92)
0
20
40
60
80
100
120
Survival time (months)
