# Supplementary material for: WDR5 Expression Is Prognostic of Breast Cancer Outcome
Source: PLoS One. 2015 Sep 10;10(9):e0124964. doi: 10.1371/journal.pone.0124964 (PMC4565643; doi:10.1371/journal.pone.0124964)
Supplement: Supplementary file 3 [file pone.0124964.s002.docx]

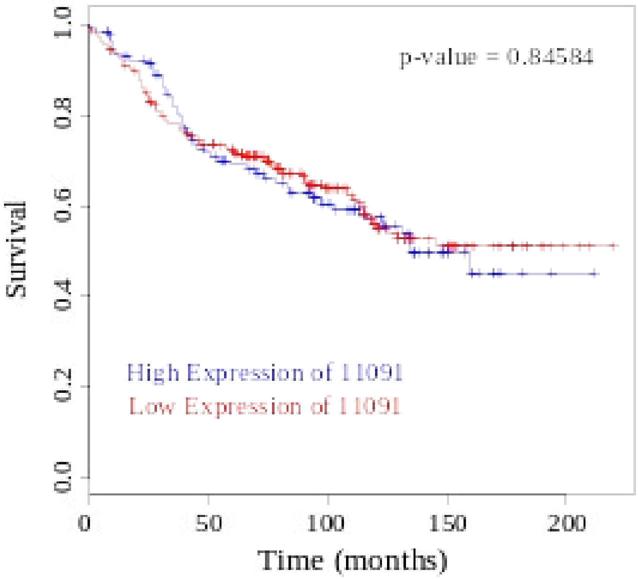
p-value = 0.84584
Survival
1.0
0.8
0.6
0.4
0.2
0.0
High Expression of 11091
Low Expression of 11091
0
50
100
150
200
Time (months)
