# Supplementary material for: WDR5 Expression Is Prognostic of Breast Cancer Outcome
Source: PLoS One. 2015 Sep 10;10(9):e0124964. doi: 10.1371/journal.pone.0124964 (PMC4565643; doi:10.1371/journal.pone.0124964)
Supplement: Supplementary file 4 [file pone.0124964.s003.docx]

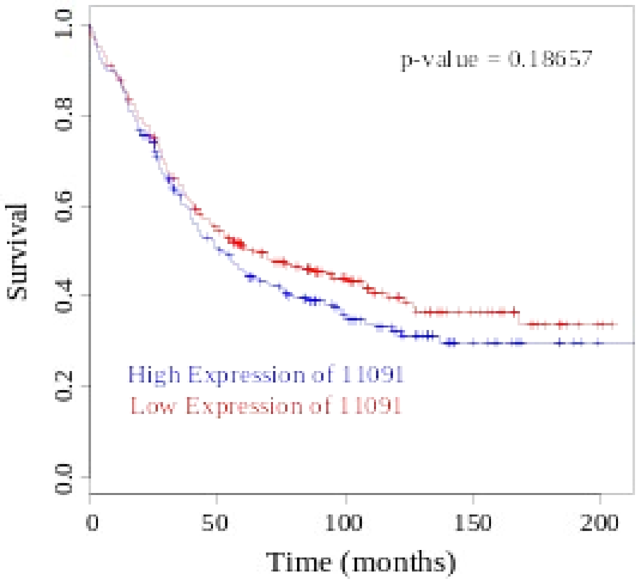

p-value = 0.18657
High Expression of 11091
Low Expression of 11091
Survival
Time (months)
1.0
0.8
0.6
0.4
0.2
0.0
0
50
100
150
200
