# Supplementary material for: WDR5 Expression Is Prognostic of Breast Cancer Outcome
Source: PLoS One. 2015 Sep 10;10(9):e0124964. doi: 10.1371/journal.pone.0124964 (PMC4565643; doi:10.1371/journal.pone.0124964)
Supplement: Supplementary file 5 [file pone.0124964.s007.docx]

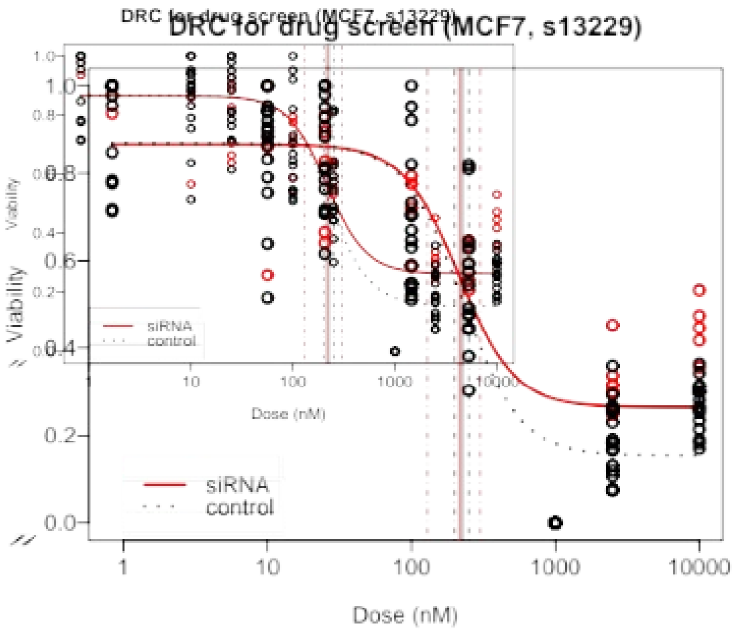
DRC for drug screen (MCF7, s13229)
Viability
Dose (nM)
siRNA
control
0.0
0.2
0.4
0.6
0.8
1.0
1
10
100
1000
10000
